# Blockade of Autophagy Prevents the Development and Progression of Peritoneal Fibrosis

**DOI:** 10.3389/fphar.2021.724141

**Published:** 2021-08-23

**Authors:** Yingfeng Shi, Yan Hu, Yi Wang, Xiaoyan Ma, Lunxian Tang, Min Tao, Andong Qiu, Shougang Zhuang, Na Liu

**Affiliations:** ^1^ Department of Nephrology, Shanghai East Hospital, Tongji University School of Medicine, Shanghai, China; ^2^ Emergency Department of Critical Care Medicine, Shanghai East Hospital, Tongji University School of Medicine, Shanghai, China; ^3^ School of Life Science and Technology, Advanced Institute of Translational Medicine, Tongji University, Shanghai, China; ^4^ Department of Medicine, Rhode Island Hospital and Alpert Medical School, Brown University, Providence, RI, United States

**Keywords:** autophagy, peritoneal fibrosis, epithelial to mesenchymal transition, profibrotic signaling pathways, inflammation

## Abstract

Peritoneal fibrosis (PF) is a major cause of ultrafiltration failure in long-term peritoneal dialysis (PD) patients. Nevertheless, limited measures have been shown to be effective for the prevention and treatment of PF. Some views reveal that activation of autophagy ameliorates PF but others demonstrate that autophagy promotes PF. It is obvious that the role of autophagy in PF is controversial and further studies are needed. Here, we investigated the role of autophagy in rat models of PF and damaged cultured human peritoneal mesothelial cells (HPMCs). Autophagy was highly activated in fibrotic peritoneum from two PF rat models induced by 4.25% peritoneal dialysate fluid (PDF) and 0.1% chlorhexidine gluconate (CG). Blockade of autophagy with 3-MA effectively prevented PF in both models and reversed epithelial to mesenchymal transition (EMT) by down-regulating TGF-β/Smad3 signaling pathway and downstream nuclear transcription factors Slug and Snail. Treatment with 3-MA also inhibited activation of EGFR/ERK1/2 signaling pathway during PF. Moreover, 3-MA prominently decreased STAT3/NF-κB-mediated inflammatory response and macrophage infiltration, and prevented peritoneal angiogenesis through downregulation of β-catenin signal. In addition, TGF-β1 stimulation up-regulated autophagic activity as evidenced by the increased autophagosome *in vitro*. Exposure of HPMCs to TGF-β1 resulted in the induction of EMT and activation of TGF-β/Smad3, EGFR/ERK1/2 signaling pathways. Treatment with 3-MA blocked all these responses. In addition, delayed administration of 3-MA was effective in reducing EMT induced by TGF-β1. Taken together, our study indicated that autophagy might promote PF and 3-MA had anti-fibrosis effect *in vivo* and *in vitro*. These results suggest that autophagy could be a potential target on PF therapy for clinical patients with long-term PD.

## Introduction

Peritoneal dialysis (PD) is a well-established alternative therapy for patients with end-stage renal disease (ESRD). According to the last registry, PD is currently used by about 11% of dialysis patients with ESRD (Jain et al., 2012). In spite of the unique advantages such as preservation of residual renal function, stable hemodynamics, and higher quality of life for patients, compared to hemodialysis, PD promotes continuous exposure of the peritoneal membrane to bioincompatible dialysis solutions, which leads to the alteration of normal peritoneal membrane structure and loss of ultrafiltration function, eventually causes the progressive development of peritoneal fibrosis (PF) and forces patients to withdraw from PD (Krediet and Struijk, 2013; Zhou et al., 2016a; Krediet, 2018). Therefore, exploring the mechanism of PF and effective prevention strategies are urgently needed.

Epithelial to mesenchymal transition (EMT) of peritoneal mesothelial cells (PMCs), also known as mesothelial to mesenchymal transition (MMT), is considered to be an important initiating factor in PF (Strippoli et al., 2016). Mounting evidence has demonstrated that transforming growth factor β1 (TGF-β1)-induced EMT is a pivotal process of progressive PF (Margetts et al., 2005). TGF-β1 signal exerts its biological effects through activating the phosphorylation of Smad2 and Smad3 by the type I TGF-β receptor (TGF-βRI), and then translocated into nucleus where they regulate gene transcription (Leask and Abraham, 2004). Previous study has confirmed that Smad3 knockout mice develop into mild PF by attenuating EMT and reducing collagen formation (Patel et al., 2010). Except for TGF-β1/Smad3 signaling pathway, activation of epidermal growth factor receptor (EGFR) pathway is another important mechanism during PF progress. Our research group demonstrated for the first time that EGFR and its downstream molecules participated in PF, and inhibition of EGFR blocked the development and progression of mouse PF induced by chlorhexidine gluconate (CG) (Wang et al., 2016).

Peritoneal inflammation is considered as an important event during the pathogenesis of PF (Zhang et al., 2017a). Peritoneal injury leads to the activation of signal transducer and activator of transcription 3 (STAT3) and nuclear factor kappa-B (NF-κB), promotes the release of multiple proinflammatory cytokines such as interleukin-6 (IL-6), monocyte chemoattractant protein-1 (MCP-1), tumor necrosis factor-α (TNF-α) and interleukin-1β (IL-1β) (Fan et al., 2013). NF-κB signaling pathway is also closely associated with the occurrence of EMT as well as macrophage recruitment (Kitterer et al., 2015). Macrophage has the ability to stimulate the production of connective tissue growth factor (CTGF), and has been involved in the activation of myofibroblasts (Wynn and Vannella, 2016). In addition, another important mechanism for PD-related PF is angiogenesis. The increase in angiogenesis is related to an increase in solute transport rate and a decline in the ultrafiltration capacity (Davies, 2004). The role of WNT/β-catenin signaling in peritoneal membrane angiogenesis has been recently clarified (Padwal et al., 2017; Padwal et al., 2018). Treatment with ICG-001, a β-catenin inhibitor, improved peritoneal angiogenesis in a mouse model of PF, and decreased levels of vascular endothelial growth factor (VEGF) (Padwal et al., 2018).

Autophagy is a self-protection mechanism to maintain the homeostasis of cellular environment, which is mediated by lysosome to degrade damaged proteins or organelles (Saha et al., 2018). Under physiological conditions, autophagy plays an vital role in ensuring the normal proliferation, differentiation and apoptosis of cells (Saha et al., 2018). However, autophagy is also involved in the pathophysiological mechanism of multiple diseases. Recent reports have shown that autophagy is closely related to pathogenesis and development of tissue fibrosis, such as heart (Zhao et al., 2018), liver (Meng et al., 2018), lung (Cabrera et al., 2015), and kidney (Zhao et al., 2019). For PF, autophagy has both positive and negative effects. The *in vitro* study demonstrated that initiation of autophagy could block the NLRP3-IL-1β mediated inflammasome activation, thus resisting the damage of high glucose (HG)-based peritoneal dialysis fluids on PMCs (Wu et al., 2016a). Only two animal experiments confirmed the protective role of autophagy against PF, and that autophagy down-regulation was observed in peritoneum from a HG-induced mouse peritoneal injury model (Yang et al., 2017; Li et al., 2019a). On the contrary, another research found that HG peritoneal dialysate induced autophagy in human peritoneal mesothelial cells (HPMCs), associated with fibrosis and apoptosis hallmarks (Wu et al., 2018). They indicated that autophagy promoted fibrosis and apoptosis in the peritoneum during long-term PD (Wu et al., 2018). Such dual character of autophagy has also been proved in renal fibrosis and hepatic fibrosis. Obviously, the role of autophagy in PF is still controversial, and it is very meaningful to conduct autophagy research on the progression of PF.

In this study, we examined the activation of autophagy in fibrotic peritoneum from two PF rat models injected with 4.25% peritoneal dialysate fluid (PDF) for 28 days and 0.1% CG for 21 days, and in TGF-β1-stimulated HPMCs. We also evaluated the effect of autophagy inhibition with 3-Methyladenine (3-MA) on fibrosis pathological changes *in vivo* and *in vitro*. Moreover, we clarified the mechanisms by which blockade of autophagy prevents the development of PF. This study elucidated the exact role and mechanism of autophagy in PF, and suggested that autophagy could be a potential target on PF therapy for clinical long-term PD patients.

## Materials and Methods

### Antibodies and Reagents

3-MA was purchased from Selleckchem (Houston, TX, United States). Antibodies to Beclin-1 (#3738), p-Smad3 (#9520), Smad3 (#9523), E-cadherin (#14472), p-EGFR (#3777), EGFR (#4267), p-ERK1/2 (#4370), ERK1/2 (#4695), p-STAT3 (#9138), STAT3 (#9139), β-actin (#4970) and Snail (#3879) were purchased from Cell Signaling Technology (Danvers, MA, United States). Antibodies to GAPDH (sc-32233), Collagen I (sc-28654), TGF-βRI (sc-399), p-NF-κB (sc-166748), NF-κB (sc-8008), IL-1β (sc-52012), CD68 (sc-20060), CD31 (sc-376764), VEGF (sc-7269) were purchased from Santa Cruz Biotechnology (San Diego, CA, United States). Antibodies to Fibronectin (ab2413) and Slug (ab27568) were purchased from Abcam (Cambridge, MA, United States). Antibody to LC3 (NB100-2220) was purchased from Novus Biologicals (Littleton, CO). Antibody to β-catenin (610154) was purchased from BD Biosciences (San Diego, CA). Antibodies to CD34 (GB13013) and IL-6 (GB11117) were purchased from Servicebio (Wuhan, China). TGF-β1, IL-1β, MCP-1 enzyme-linked immunosorbent assay (ELISA) kits and TGF-β1 protein were purchased from R&D Systems (Minneapolis, MN, United States). Beclin-1 siRNA was purchased from GenePharma (Shanghai, China). Lipofectamine 2000 was purchased from Invitrogen (Grand Island, NY, United States). 4.25% glucose peritoneal dialysis solution was purchased from Baxter Healthcare (Guangzhou, China). Antibody to α-SMA (A2547), chlorhexidine gluconate, DMSO and all other chemicals were obtained from Sigma-Aldrich (St. Louis, MO, United States).

### Animal Models and Experimental Design

Male Sprague-Dawley rats (Shanghai Super-B&K Laboratory Animal Corp. Ltd., Shanghai, China) that weighed 200–220 g were housed under a 12 h light-dark cycle with food and water supplied ad libitum at the Experimental Animal Center of Tongji University. Two PF rat models were established respectively by intraperitoneal injection of 4.25% high glucose PDF (100 ml/kg) every day for 28 days (Xu et al., 2017; Shi et al., 2020) and 0.1% CG (10 ml/kg) dissolved in saline every other day for 21 days (Kokubo et al., 2012; Io et al., 2015; Shi et al., 2020). To investigate the anti-fibrosis effect of 3-MA on PF, rats were injected intraperitoneally with two doses of 3-MA (15 and 30 mg/kg) in warmed saline every day. 3-MA is administrated apart from the 10 ml/kg of CG or 100 ml/kg of PDF. Rats were randomly divided into four groups in each model: 1) Rats injected with an equivalent amount of saline intraperitoneally, defined as sham group (*n* = 6); 2) Rats injected with an equivalent amount of saline intraperitoneally and 3-MA, defined as sham + 3-MA group (*n* = 6); 3) Rats injected with 4.25% PDF or 0.1% CG and an equivalent amount of saline intraperitoneally, defined as PDF/CG group (*n* = 6); 4) Rats injected with 4.25% PDF or 0.1% CG and 3-MA, defined as PDF/CG+3-MA group (*n* = 6). At the end of 28 or 21 days, all rats were killed by exsanguination under anesthesia with inhaled 5% isoflurane in room air and the parietal peritoneum was collected from each group for further protein analysis and histological examination. The animal protocol was reviewed and approved by the Institutional Animal Care and Use Committee at Tongji University.

### Cell Culture and Treatments

HPMCs (kind gifts from Haiping Mao at Sun Yat-Sen University, Guangzhou, China) were cultured in MEM medium containing 10% fetal bovine serum (FBS), 1% penicillin and streptomycin in an atmosphere of 5% CO2 and 95% air at 37°C. We passed the primary cells for three generations, obtained a stable phenotype, and then started the formal experiments. To examine the inhibitory effect of 3-MA on TGF-β1-induced EMT *in vitro*, subconfluent HPMCs were starved for 24 h in MEM medium containing 0.5% FBS and then exposed to TGF-β1 (2 ng/ml) in the presence of 3-MA (0, 1, 5 and 10 mM) for 36 h. After exposed for 36 h, cells were harvested for immunoblotting analyses and transmission electron microscope observation. To determine the effects of delayed treatment of 3-MA on TGF-β1-induced EMT, subconfluent HPMCs were starved for 24 h in MEM medium containing 0.5% FBS followed by stimulation with TGF-β1 (2 ng/ml) for 48 h and then incubated with 10 mM 3-MA for an additional 24 or 48 h, cells were harvested for immunoblotting analyses. All of the *in vitro* experiments were repeated for at least three times.

### siRNA Transfection

The small interfering (si) RNA oligonucleotides targeted specially for Beclin-1 and non-targeting control siRNA were used in this study. The Beclin-1 siRNA (sense: 5ʹ-CAG​UUU​GGC​ACA​AUC​AAU​ATT-3ʹ and antisense: 5ʹ-UAU​UGA​UUG​UGC​CAA​ACU​GTT-3ʹ) were chemically synthesized by GenePharma (Shanghai, China). HPMCs were seed to 30–40% confluence in antibiotic-free medium and cultured for 24 h then transfected with Beclin-1 siRNA (100 pmol) with lipofectamine 2000 (Invitrogen, Grand Island, NY, United States) according to the manufacturer’s instructions. In parallel, scrambled siRNA (100 pmol) was used as control for off-target changes in HPMCs. After transfection for 24 h, the original antibiotic-free medium was changed and cells were treated with TGF-β1 (2 ng/ml) for an additional 36 h before being harvested for the further experiments.

### ELISA Analysis

ELISA detection of TGF-β1, IL-1β and MCP-1 proteins in peritoneal tissue from each group were performed in accordance with the manufacturer’s instructions.

### Immunoblot Analysis

Immunoblot analysis of rat peritoneal tissue and cell samples was conducted as described previously (Zhou et al., 2016b). The densitometry analysis of immunoblot results was conducted by using ImageJ software.

### Morphologic Studies of Peritoneum

Formalin-fixed peritoneum was embedded in paraffin and cut into 3-μm-thick sections. For evaluation of PF, Masson’s trichrome staining and Sirius red staining were performed according to the protocol provided by the supplier (Sigma-Aldrich). The positive area and thickness of the submesothelial tissue were measured, and the average of ten independent measurements were calculated for each section.

### Immunohistochemical Staining

Sections cut at 3 μm thick were de-paraffinized and rehydrated, quenched with 3% H_2_O_2_, immersed in citrate buffer and heated in a microwave for retrieval of antigens as described in our previous study (Pang et al., 2009). Slides were viewed with a Nikon Eclipse 80i microscope equipped with a digital camera (DS-Ri1, Nikon, Shanghai, China).

### Immunofluorescence Staining

Immunofluorescence staining was carried out according to the procedure described in our previous study (Pang et al., 2009). Formalin-Fixed Paraffin-Embedded (FFPE) sections (3 μm) were rehydrated and incubated with primary antibodies against Beclin-1, E-cadherin or Snail and then Texas Red- or FITC-labeled secondary antibodies (Invitrogen).

### Transmission Electron Microscope

Transmission electron microscope (TEM) was performed to observe the morphology of autophagosome. After indicated treatments, cells were collected from each group for standard TEM processing. And various autophagic structures including phagophore, autophagosome, and autolysosome in HPMCs were revealed at high magnification from each cell and digital images with scale bars were taken.

### Statistical Analysis

Data depicted in graphs represent the means ± SEM for each group. Intergroup comparison was made using one-way ANOVA. Multiple means were compared using Tukey’s test. The differences between two groups were determined by Student’s t-test. Statistically significant differences between mean values were marked in each graph. *p* < 0.05 was considered significant. The statistical analyses were conducted by using IBM SPSS Statistics 20.0 (Version X; IBM, Armonk, NY, United States).

## Results

### Administration of 3-MA Inhibits Autophagy and Prevents PF in Both 4.25% PDF-Induced Rat Model and 0.1% CG-Induced Rat Model

**FIGURE 1:**
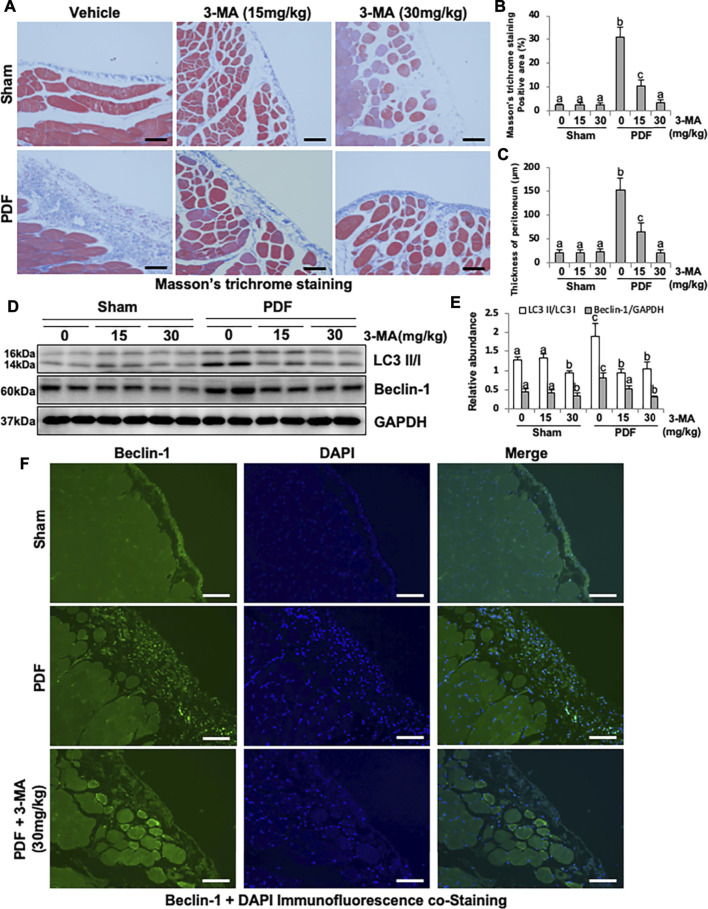
Administration of 3-MA inhibits autophagy and prevents peritoneal fibrosis in 4.25% PDF-induced rat model. **(A)** Photomicrographs show Masson’s trichrome staining of the peritoneum in each group. **(B)** Positive area of the Masson-positive submesothelial area (blue). **(C)** Thickness of the Masson-positive submesothelial area (blue). **(D)** Peritoneum tissue lysates were subjected to immunoblot analysis with specific antibodies against LC3, Beclin-1 and GAPDH. **(E)** Expression levels of LC3II and Beclin-1 were quantified by densitometry and normalized with LC3I and GAPDH respectively. **(F)** Photomicrographs show immunofluorescence co-staining of Beclin-1 and DAPI. Data are represented as the mean ± SEM. Means with different superscript letters are significantly different from one another (*p* < 0.05). All scale bars = 100 μm.

To elucidate the role of autophagy in the development of PF, we first established a model of PF in rats by intraperitoneal injection with 4.25% high glucose peritoneal dialysate for 28 days and concurrently given 3-MA treatment (15 and 30 mg/kg). As shown in Figure 1, thickening of the submesothelial compact zone is demonstrated by Masson’s trichrome staining. Treatment with 3-MA prevents PF in a dose-dependent manner as indicated by the significant reduction of Masson’s trichrome staining positive area and the thickness of peritoneum (Figures 1A–C). Low homeostasis level of autophagy was detected in the normal rats with/without 3-MA injection, but it was significantly increased in the PF rats, as evidenced by the high level of LC3B II to I ratio and Beclin-1. Both dose of 3-MA could inhibit the anomalous up-regulation of autophagy caused by high glucose dialysate, as indicated in the decreased of LC3B II to I ratio and Beclin-1 (Figures 1D,E). Moreover, immunofluorescence co-staining showed that Beclin-1 was highly expressed in thickened peritoneum from PF rats, and mainly located in peritoneal mesothelial cells (Figure 1F).

Next, we established another rat model of PF by intraperitoneal injection of 0.1% CG every other day for 21 days, so as to verify the anti-peritoneal fibrosis effect of 3-MA again. Similarly, thickening of the submesothelial compact zone is demonstrated by the Masson’s trichrome staining and Sirius red staining in PF rats, administration of 3-MA (30 mg/kg) significantly decreased thickness of the fibrotic submesothelial area and the quantified positive staining area (Supplementary Figures S1A–D). Consistent with PDF-model, increased expression of LC3 and Beclin-1 were observed in CG-induced PF rats, treatment with 3-MA remarkably blocked its expression (Supplementary Figures S1E,F). Taken together, these results indicate that autophagy is up-regulated in peritoneum in both PF models and 3-MA has the ability to prevent PF.

### Blockade of Autophagy With 3-MA Prevents EMT in Both PF Rat Models

**FIGURE 2:**
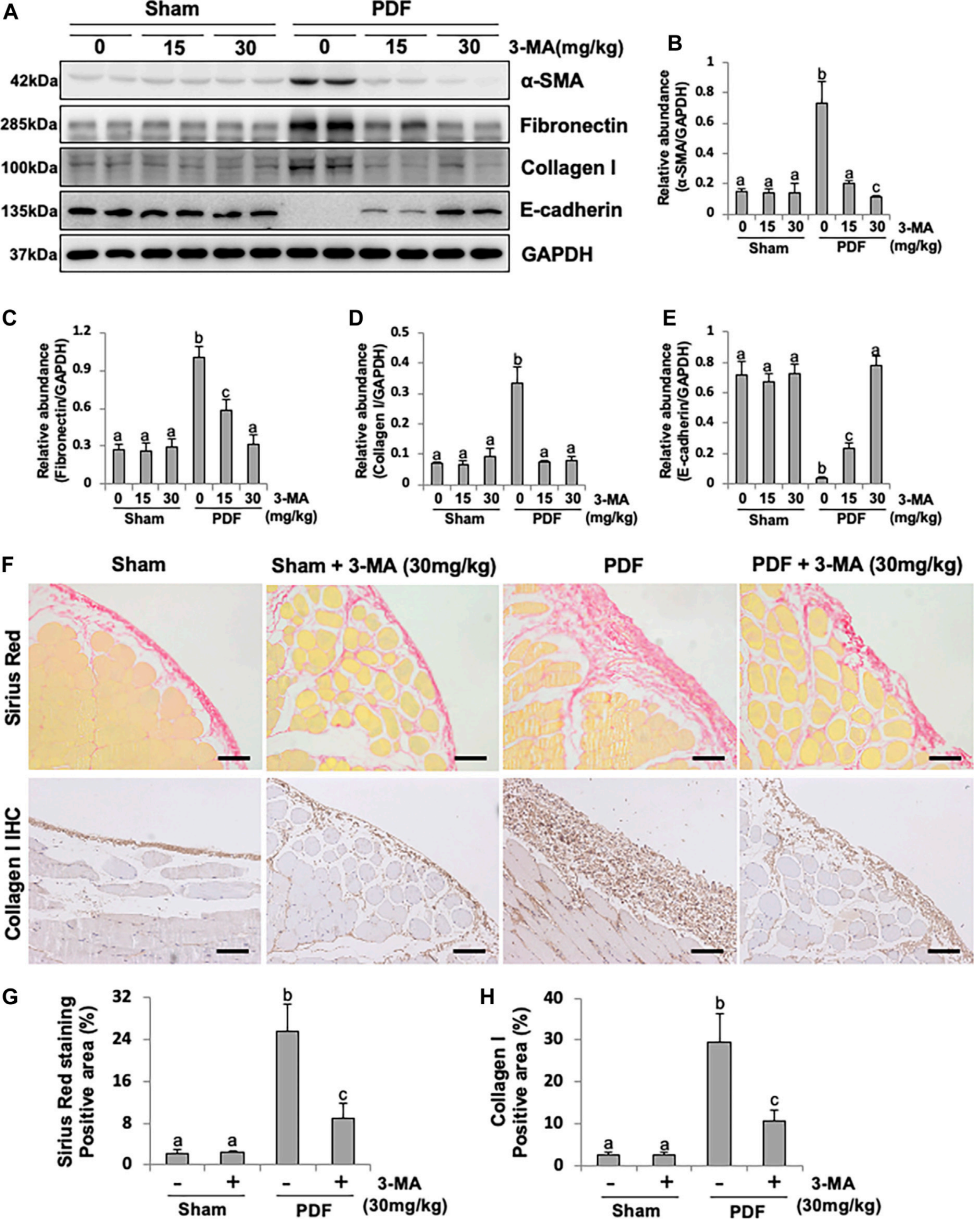
Blockade of autophagy with 3-MA prevents EMT in a peritoneal fibrosis rat model. **(A)** Peritoneum tissue lysates were subjected to immunoblot analysis with specific antibodies against α-SMA, Fibronectin, Collagen I, E-cadherin and GAPDH. **(B**–**E)** Expression levels of α-SMA, Fibronectin, Collagen I and E-cadherin were quantified by densitometry and normalized with GAPDH. **(F)** Photomicrographs illustrate Sirius Red staining and immunohistochemistry staining of Collagen I from peritoneal tissues. **(G)** Positive area of Sirius Red-positive submesothelial area. **(H)** Positive area of Collagen I. Data are represented as the mean ± SEM. Means with different superscript letters are significantly different from one another (*p* < 0.05). All scale bars = 100 μm.

EMT occurs after peritoneal injury and is involved in the development of PF (Liu et al., 2015). To understand whether autophagy mediates PF by regulating the process of EMT, we examined the effect of 3-MA in both PF rat models. As shown in Figures 2A–E and Supplementary Figure S2, injection of PDF or CG caused a significant reduction of the protein levels of E-cadherin, an epithelial cell marker, and increased expression of α-SMA, Fibronectin, and Collagen I, three mesenchymal markers. Administrated with 3-MA in both dose of 15 mg/kg and 30 mg/kg could down-regulate the expression levels of three mesenchymal marker as mentioned above (Figures 2A–D). Moreover, 3-MA effectively restored the expression of E-cadherin in the damaged peritoneum, especially for the 30 mg/kg treatment group, whose expression level was similar to that of the normal rats (Figures 2A,E). In addition, both of Sirius red staining and Collagen I immunohistochemical staining further confirmed that 3-MA inhibited collagen formation and accumulation of the submesothelial compact zone, and markedly reduced quantitative positive area of them (Figures 2F–H). These data suggest that 3-MA prevents PF by inhibiting the process of EMT.

### Inhibition of Autophagy With 3-MA Blocks EMT in PF Rats by Regulating TGF-β/Smad3 Signaling Pathway and Downstream Nuclear Transcription Factors: Slug and Snail

**FIGURE 3:**
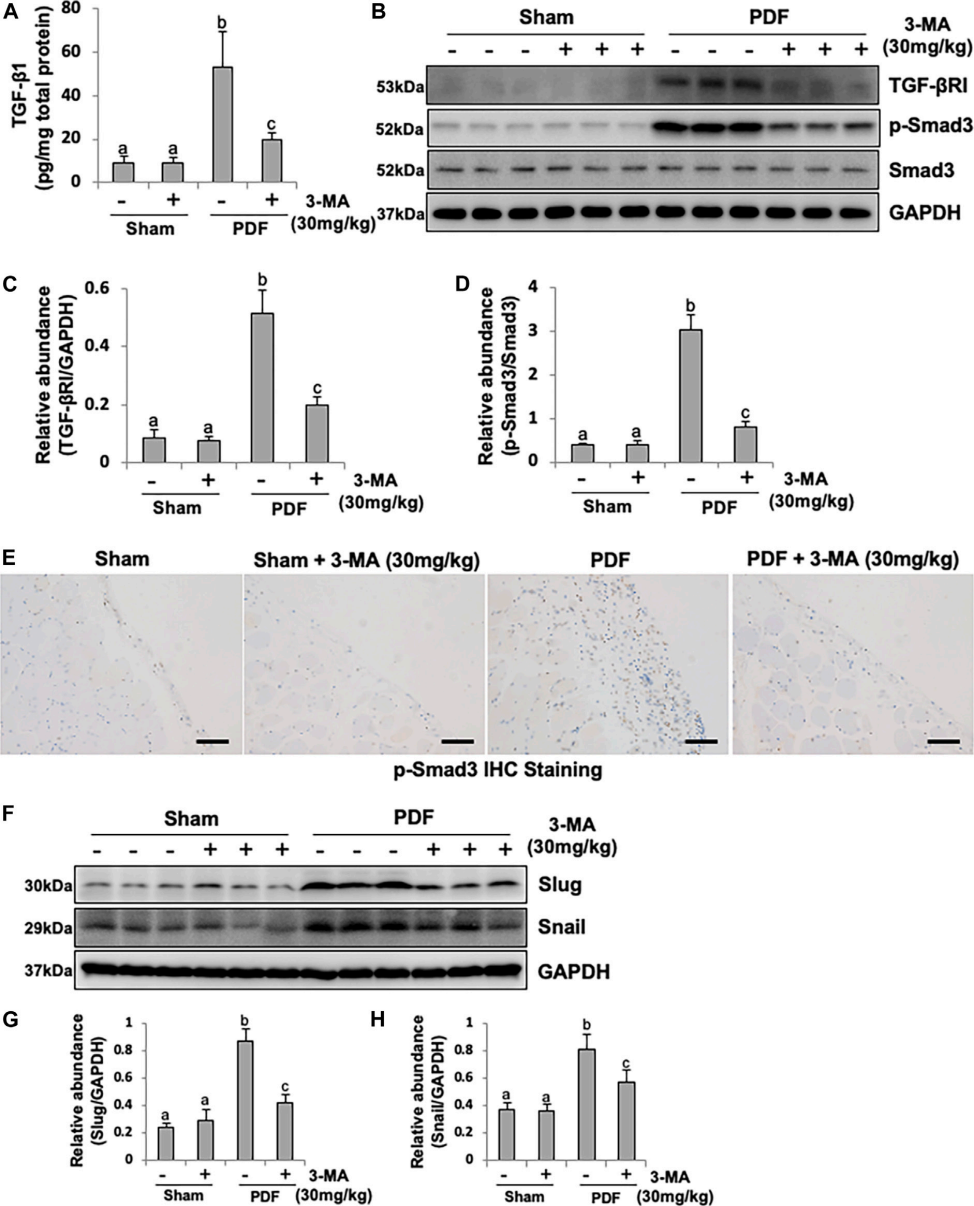
Inhibition of autophagy with 3-MA blocks EMT in PF rats by regulating TGF-β/Smad3 signaling pathway and downstream nuclear transcription factors: Slug and Snail. **(A)** TGF-β1 level in peritoneum from each group by ELISA kit detection. **(B)** Peritoneum tissue lysates were subjected to immunoblot analysis with specific antibodies against TGF-βRI, p-Smad3, Smad3 and GAPDH. **(C)** Expression level of TGF-βRI was quantified by densitometry and normalized with GAPDH. **(D)** Expression level of p-Smad3 was quantified by densitometry and normalized with Smad3. **(E)** Photomicrographs illustrate immunohistochemistry staining of p-Smad3 from peritoneal tissues. **(F)** Peritoneum tissue lysates were subjected to immunoblot analysis with specific antibodies against Slug, Snail and GAPDH. **(G, H)** Expression levels of Slug and Snail were quantified by densitometry and normalized with GAPDH. Data are represented as the mean ± SEM. Means with different superscript letters are significantly different from one another (*p* < 0.05). All scale bars = 100 μm.

The production of TGF-β1, stimulated by high glucose and acid pH, is the main extracellular matrix (ECM) and collagen-producing factor during organ fibrosis (Massagué, 2012). To demonstrated whether PDF could induce TGF-β1 production in peritoneum, the secretion of TGF-β1 from peritoneum was determined by ELISA kit. As shown in Figure 3A, higher level of TGF-β1 was observed in injured peritoneum from PDF-model group than sham rats, administrated with 3-MA significantly reduced the production of TGF-β1. To further identify autophagy might be involved in the process of EMT by regulating TGF-β/Smad3 signaling pathway, Western blotting analysis was conducted. Immunoblot analysis showed that exposure to 4.25% PDF resulted in the activation of TGF-β/Smad3 signaling pathway, as evidenced by the significant increased expression of TGF-βRI and p-Smad3. Single dose of 3-MA decreased TGF-βRI and inhibited phosphorylation of Smad3, but had no impact on total Smad3 (Figures 3B–D). Immunohistochemical staining of p-Smad3 further confirmed inhibitory effect of 3-MA on Smad3 activation (Figure 3E). Furthermore, since TGF-β/Smad3 signaling pathway directly regulates Slug and Snail, two nuclear transcriptional repressors of E-cadherin, thus promoting EMT (Cano et al., 2000), we also examined the impact of 3-MA on their expressions. Basal levels of Slug and Snail were observed in the normal rats without PDF daily injection, while their expression levels were dramatically increased in PF rats. Treatment with 3-MA restored Slug to the normal level and lowered Snail to half percent (Figures 3F–H). Therefore, 3-MA could inhibit EMT during PF through inactivation of TGF-β/Smad3 signaling pathway and E-cadherin transcriptional inhibition by down-regulating Slug and Snail.

### 3-MA Decreases Autophagic Activity in Cultured Human Peritoneal Mesothelial Cells

**FIGURE 4:**
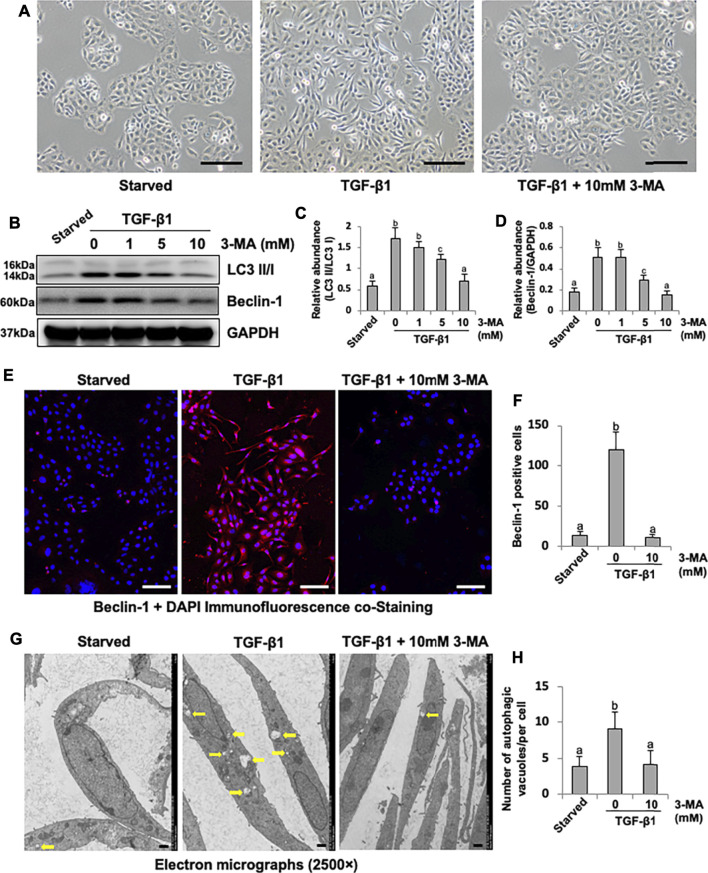
3-MA decreases autophagic activity in cultured human peritoneal mesothelial cells. **(A)** Photomicrographs illustrate light microscope observation of HPMCs. **(B)** Cell lysates were subjected to immunoblot analysis with specific antibodies against LC3, Beclin-1 and GAPDH. **(C, D)** Expression levels of LC3II and Beclin-1 were quantified by densitometry and normalized with LC3I and GAPDH respectively. **(E)** Photomicrographs show immunofluorescence co-staining of Beclin-1 and DAPI. **(F)** The count of Beclin-1-positive cells. **(G)** Transmission electron microscopy images of autophagosome (Yellow arrows) in HPMCs following TGF-β1 (2 ng/ml) stimulation in the presence/absence of 3-MA. **(H)** Quantitation of the number of autophagic vacuoles per cell. Data are represented as the mean ± SEM. Means with different superscript letters are significantly different from one another (*p* < 0.05). Scale bars in **(A)** = 500 μm, **(E)** = 100 μm, **(F)** = 10 μm.

Cultured HPMCs were stimulated by TGF-β1 and treated with 3-MA at different doses (1, 5, 10 mM). TGF-β1 was found to induce the morphological transition of HPMCs, which stimulated HPMCs to lose their epithelial shape to an elongated shape, treatment with 3-MA prevented the above morphologic changes of cultured HPMCs (Figure 4A). In addition, TGF-β1 also triggered a significant up-regulation of LC3B II to I ratio and Beclin-1. 3-MA dose-dependently suppressed these responses (Figures 4B–D). Immunofluorescence assay showed that the expression of Beclin-1 was significantly increased in response to TGF-β1, 3-MA treatment inhibited its expression (Figures 4E,F). As a gold standard to observe autophagosome, images from TEM revealed accumulation of autophagy-related vacuoles in TGF-β1-stimulated HPMCs compared to control cells. Administration with 3-MA significantly prevented the autophagic activity (Figures 4G,H). Above-mentioned results point out that autophagic activity is activated by TGF-β1 stimulation in cultured HPMCs.

### Inhibition of Autophagy With 3-MA Blocks EMT in HPMCs by Regulating TGF-β/Smad3 Signaling Pathway and Downstream Nuclear Transcription Factors: Slug and Snail

**FIGURE 5:**
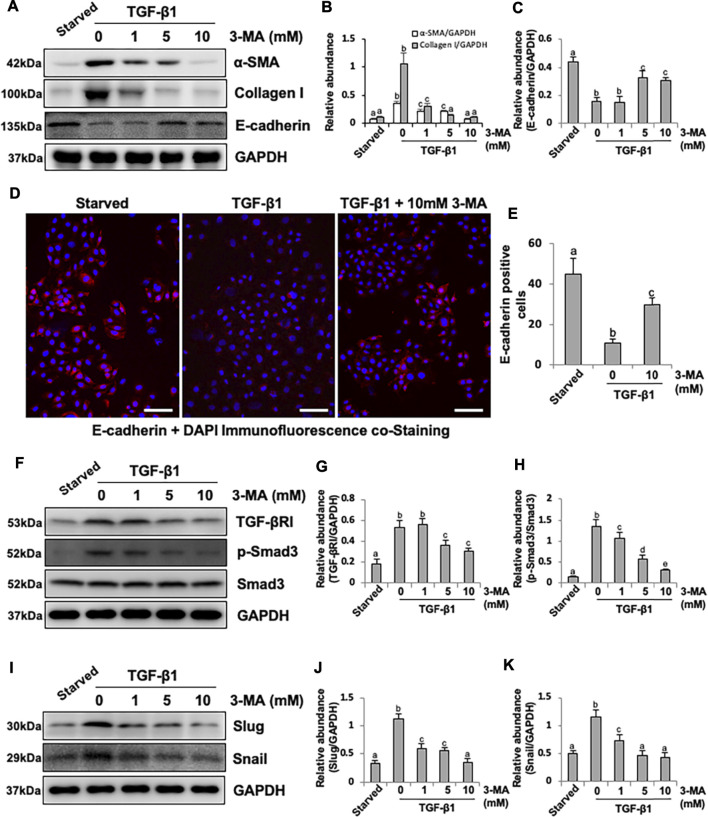
Inhibition of autophagy with 3-MA blocks EMT in HPMCs by regulating TGF-β/Smad3 signaling pathway and downstream nuclear transcription factors: Slug and Snail. **(A)** Cell lysates were subjected to immunoblot analysis with specific antibodies against α-SMA, Collagen I, E-cadherin and GAPDH. **(B, C)** Expression levels of α-SMA, Collagen I and E-cadherin were quantified by densitometry and normalized with GAPDH. **(D)** Photomicrographs show immunofluorescence co-staining of E-cadherin and DAPI. **(E)** The count of E-cadherin-positive cells. **(F, I)** Cell lysates were subjected to immunoblot analysis with specific antibodies against TGF-βRI, p-Smad3, Smad3, Slug, Snail and GAPDH. **(G)** Expression level of TGF-βRI was quantified by densitometry and normalized with GAPDH. **(H)** Expression level of p-Smad3 was quantified by densitometry and normalized with Smad3. **(J, K)** Expression levels of Slug and Snail were quantified by densitometry and normalized with GAPDH. Data are represented as the mean ± SEM. Means with different superscript letters are significantly different from one another (*p* < 0.05). All scale bars = 100 μm.

Since our previous studies have proved that TGF-β1 stimulation could cause EMT of HPMCs, which imitates the early stage of PMC injury during PD process (Xu et al., 2017; Shi et al., 2020), we also examined the effect of 3-MA on the EMT of cultured HPMCs in response to TGF-β1. Exposure of HPMCs to TGF-β1 at 2 ng/ml resulted in increased expression of α-SMA and Collagen I and decreased expression of E-cadherin, administrated with 3-MA inhibited TGF-β1-induced upregulation of α-SMA and Collagen I and downregulation of E-cadherin in a dose dependent manner (Figures 5A–C). Immunofluorescence assay further confirmed that the expression of E-cadherin was significantly reduced in response to TGF-β1, treatment with 3-MA increased the level of E-cadherin (Figures 5D,E). Mechanistically, 3-MA significantly reduced the expression of TGF-βRI and inhibited Smad3 phosphorylation, but made no difference on total Smad3 (Figures 5F–H). Additionally, 3-MA also down-regulated two nuclear transcription factors, Slug and Snail, involved in TGF-β1/Smad3-related EMT (Figures 5I–K). In summary, these results reiterate the inhibitory effect of 3-MA on the process of EMT by regulating TGF-β/Smad3 signaling pathway and downstream nuclear transcription factors *in vitro*.

Furthermore, to determine the effects of delayed treatment of 3-MA on TGF-β1-induced EMT, we designed a treatment scheme as shown in Supplementary Figure S3A. At 24 h after pretreatment with TGF-β1, expression of α-SMA and Collagen I was induced, and further increased at 48 h. The expression of E-cadherin decreased at 24 h and further inhibited at 48 h. However, delayed treatment with 3-MA suppressed further increases of α-SMA and Collagen I and decreases of E-cadherin (Supplementary Figures S3B–E). These data suggest that delayed administration of 3-MA is effective in reducing EMT induced by TGF-β1.

Since 3-MA is not a specific autophagy inhibitor, specifically, 3-MA is a generic inhibitor of PI3Ks. To determine whether 3-MA effects on autophagy dominating among others, we downregulated the expression of the autophagic gene, Beclin-1 by using Beclin-1 siRNA in HPMCs to investigate the effect on TGF-β/Smad3, EGFR/ERK1/2 and STAT3/NF-κB signaling pathways. As shown in Supplementary Figure S4, reduction of Beclin-1 expression by its specific siRNA decreased TGF-β1 stimulated expression of α-SMA and Collagen I (Supplementary Figures S4A,B). Mechanistically, downregulation of Beclin-1 resulted in decreased of expression of p-Smad3, p-ERK1/2, and p-STAT3 without alteration of total Smad3, ERK1/2, and STAT3 (Supplementary Figures S4C–H). However, downregulation of Beclin-1 had no impact on p-EGFR and p- NF-κB (Data not show). It is reported that EGFR signaling can be activated by ATG genes (Zhang et al., 2019), we speculate that downregulation of ATG genes in HPMCs may affect the expression of p-EGFR and p-NF-κB. Collectively, these data suggest that inhibition of Beclin-1 by siRNA can remarkably inhibited TGF-β1 induced activation of Smad3/ERK1/2/STAT3 pathway in HPMCs.

### 3-MA Inhibits Activation of EGFR/ERK1/2 Signaling Pathway *in vivo* and *in vitro*

**FIGURE 6:**
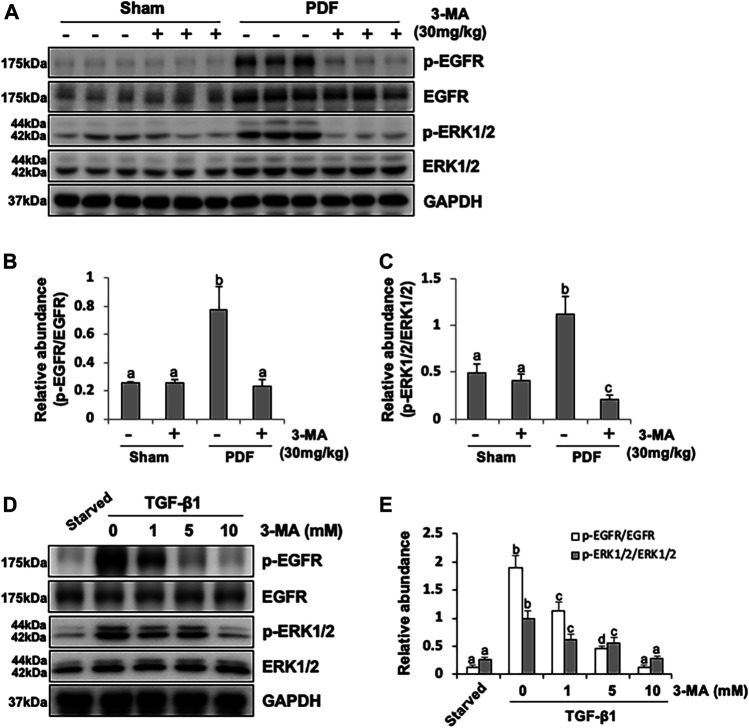
3-MA inhibits activation of EGFR/ERK1/2 signaling pathway *in vivo* and *in vitro*. **(A)** Peritoneum tissue lysates were subjected to immunoblot analysis with specific antibodies against p-EGFR, EGFR, p-ERK1/2, ERK1/2 and GAPDH. **(B, C)** Expression levels of p-EGFR and p-ERK1/2 were quantified by densitometry and normalized with EGFR and ERK1/2 respectively. **(D)** Cell lysates were subjected to immunoblot analysis with specific antibodies against p-EGFR, EGFR, p-ERK1/2, ERK1/2 and GAPDH. **(E)** Expression levels of p-EGFR and p-ERK1/2 were quantified by densitometry and normalized with EGFR and ERK1/2 respectively. Data are represented as the mean ± SEM. Means with different superscript letters are significantly different from one another (*p* < 0.05).

Besides TGF-β/Smad3 signaling pathway involved in PF, EGFR together with its downstream signal molecule ERK1/2 are also participated in the development of fibrogenesis. Thus, we set out to examined the effect of 3-MA on the activation of EGFR and ERK1/2 by immunoblotting assays. As shown in Figures 6A–C, phosphorylated EGFR and ERK1/2 were largely increased in the peritoneal membrane exposure to PDF. Inhibition of autophagy down-regulated phosphorylation of EGFR and decreased the ratio between p-EGFR and EGFR, as well as p-ERK1/2 and ERK1/2. Both total EGFR and ERK1/2 were increased after peritoneal injury, but not affected by 3-MA administration (Figures 6A–C). Moreover, we further examined whether EGFR/ERK1/2 signaling pathway would play an important role in TGF-β1-stimulated HPMCs *in vitro*. Exposure of HPMCs to TGF-β1 induced expression of p-EGFR and p-ERK1/2, treatment with 3-MA blocked all these responses, but had no impact on the expression of total EGFR and total ERK1/2 (Figures 6D,E). Collectively, these data demonstrate that 3-MA may prevent PF by inactivation of EGFR/ERK1/2 signaling pathway.

### 3-MA Prevents Inflammation and Macrophage Infiltration in PDF-Induced PF Rats

**FIGURE 7:**
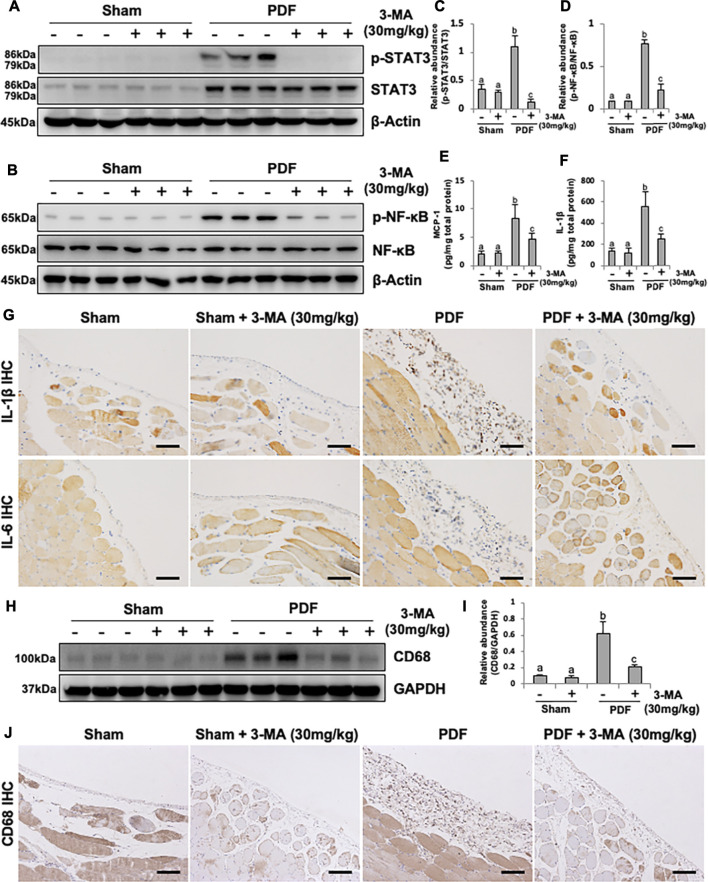
3-MA prevents inflammation and macrophage infiltration in PDF-induced PF rats. **(A, B)** Peritoneum tissue lysates were subjected to immunoblot analysis with specific antibodies against p-STAT3, STAT3, p-NF-κB, NF-κB and β-actin. **(C, D)** Expression levels of p-STAT3 and p-NF-κB were quantified by densitometry and normalized with STAT3 and NF-κB respectively. **(E, F)** The levels of MCP-1 and IL-1β in peritoneum from each group were detected by ELISA kit. **(G)** Photomicrographs illustrate immunohistochemistry staining of IL-1β and IL-6 from peritoneal tissues. **(H)** Peritoneum tissue lysates were subjected to immunoblot analysis with specific antibodies against CD68 and GAPDH. **(I)** Expression level of CD68 was quantified by densitometry and normalized with GAPDH. **(J)** Photomicrographs illustrate immunohistochemistry staining of CD68 from peritoneal tissues. Data are represented as the mean ± SEM. Means with different superscript letters are significantly different from one another (*p* < 0.05). All scale bars = 100 μm.

Considering the pivotal role of inflammation in PF, we further explored the anti-inflammatory effect of 3-MA. STAT3 and NF-κB-induced inflammatory signaling pathways were evidently activated after exposure to non-biocompatible peritoneal dialysate for 28 days. Combination therapy with 3-MA down-regulated phosphorylated STAT3 and NF-κB (Figures 7A–D). The phosphorylation of NF-κB triggers the release of large number of inflammatory factors such as MCP-1, IL-1β and IL-6 (Choi et al., 2017). As a result, we further evaluated the effect of 3-MA on the expression of some inflammatory cytokines by ELISA kit analysis and immunohistochemical staining. As demonstrated in Figures 7E–G, all levels of these inflammatory factors were increased in the peritoneum of rats administrated with 4.25% PDF and inhibited by 3-MA treatment. In addition, increased macrophage infiltration in the peritoneal membrane was also involved in PF (Wang et al., 2016). To explore whether autophagy was involved in this process, we examined the level of CD68, one of macrophage markers, by conducting immunoblot analysis. Injection of 4.25% PDF significantly increased the expression of CD68, treatment with 3-MA effectively decreased its expression (Figures 7H,I). Furthermore, we examined CD68 expression by immunohistochemistry and showed that the number of CD68 positive cells was elevated in the peritoneum exposure to PDF and largely reduced after 3-MA treatment (Figure 7J). Thus, these results demonstrated that inflammation inhibition is also one of the mechanisms by which 3-MA prevents PF.

### 3-MA Suppresses Peritoneal Angiogenesis Through Inhibiting β-Catenin Signaling Pathway

**FIGURE 8:**
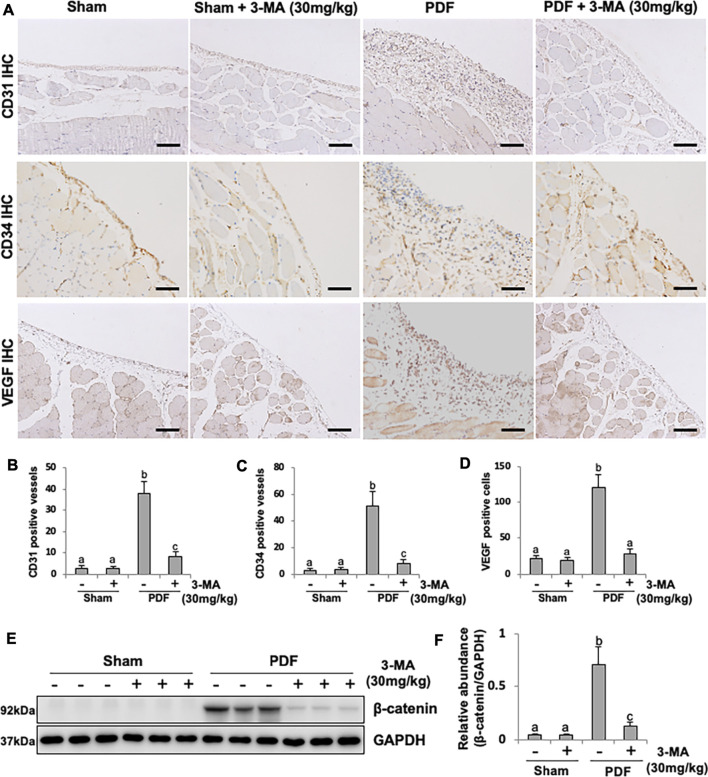
3-MA suppresses peritoneal angiogenesis through inhibiting β-catenin signaling pathway. **(A)** Photomicrographs illustrate immunohistochemistry staining of CD31, CD34 and VEGF from peritoneal tissues. **(B-D)** The count of CD31-positive vessels, CD34-positive vessels and VEGF-positive cells. **(E)** Cell lysates were subjected to immunoblot analysis with specific antibodies against β-catenin and GAPDH. **(F)** Expression level of β-catenin was quantified by densitometry and normalized with GAPDH. Data are represented as the mean ± SEM. Means with different superscript letters are significantly different from one another (*p* < 0.05). All scale bars = 100 μm.

Angiogenesis is a critical change of peritoneal structure in long term PD patients, which is associated with ultrafiltration failure (Shi et al., 2017). VEGF acted directly on the vascular endothelial cell mitogen, leading to the formation of new blood vessels. To further understand whether autophagy is involved in peritoneal angiogenesis, we examined the effect of 3-MA on PF rats. Exposure of peritoneum to 4.25% PDF promoted peritoneal angiogenesis as evidenced by the up-regulation of CD31, CD34 and VEGF, three common markers of blood vessels. Administration of 3-MA could blockade this pathological change and decrease the number of CD31-and CD34-positive vessels, as well as VEGF-positive endothelial cells (Figures 8A–D). Mechanistically, it was reported that β-catenin signaling pathway plays an important role in peritoneal angiogenesis (Padwal et al., 2018). Our immunoblotting analysis demonstrated that β-catenin pathway was dramatically activated in PF rats, treatment with 3-MA remarkably decreased the expression of β-catenin in fibrotic peritoneum (Figures 8E,F). Taken together, these data suggest that 3-MA suppresses peritoneal angiogenesis by inhibiting β-catenin signaling pathway.

**FIGURE 9:**
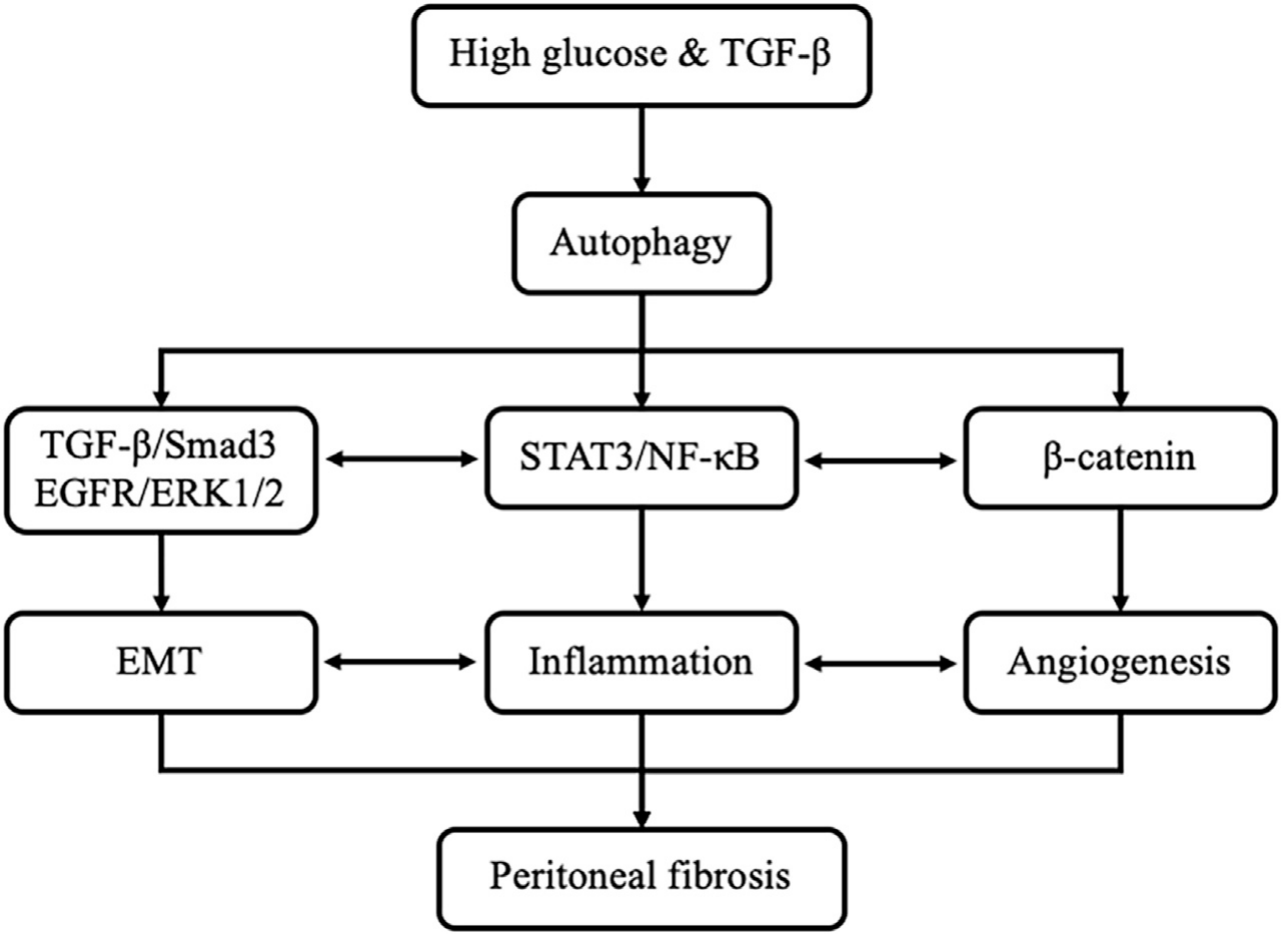
Signaling pathways of autophagy promotes PF. Exposure of high glucose or TGF-β can induce the activation of autophagy *in vivo* and *in vitro*, respectively. Activation of autophagy leads to EMT, induction of proinflammatory responses and triggering angiogenesis by regulating TGF-β/Smad3, EGFR/ERK1/2, STAT3/NF-κB and β-Catenin axis, eventually caused peritoneal fibrosis. In addition, EMT also implicated in inflammation and angiogenesis. Inflammatory responses also induce EMT and angiogenesis.

Actually, the mechanisms of autophagy promoting peritoneal fibrosis are complicated. In the present study, we found that high glucose and TGF-β can stimulate the activation of autophagy. Inhibition of autophagy with 3-MA prevents peritoneal fibrosis by regulating TGF-β/Smad3, EGFR/ERK1/2, STAT3/NF-κB and β-Catenin axis both *in vivo* and *in vitro* systems. Interestingly, a previous study shown that TGF-β induces EMT, which is also implicated in inflammation and angiogenesis due to the cytokines released by mesothelial cells that suffered EMT after exposition to glucose or TGF-β. Inflammatory cells recruited to the peritoneal cavity also induce EMT and angiogenesis (Tzavlaki and Moustakas, 2020). It can be seen that the crosstalk and interplay among multiple pathways associated with autophagy and peritoneal fibrosis, such as EMT, inflammation, and angiogenesis are complicated (Figure 9).

## Discussion

PF is one of the most common complications for long-term PD patients (Zhou et al., 2016; Krediet, 2018). At present, there is no effective intervention and treatment strategies for PF. Autophagy is a cellular process of the formation of autophagosomes by bulk degradation of cytoplasmic components. In addition to its bona fide function of catabolism, autophagy also plays important roles in fibrotic diseases (Saha et al., 2018). The role of autophagy in PF remains largely unclear and the findings from recent researches are inconsistent and very controversial (Yang et al., 2017; Wu et al., 2018; Li et al., 2019a). Using pharmacological inhibitory approach, our study has determined the regulation of PF by autophagy in two PF rat models induced by 4.25% PDF and 0.1% CG and *in vitro* model of TGF-β1 stimulated HPMCs. Blockade of autophagy by pharmacological inhibitor 3-MA effectively prevented PF by inhibiting EMT and the activation of TGF-β/Smad3, EGFR/ERK1/2 signaling pathways. 3-MA also prevented inflammation and macrophage infiltration by regulating the STAT3 and NF-κB signaling pathway. Moreover, inhibition of autophagy blockaded peritoneal angiogenesis in the injured peritoneum. In consistent with Wu et al. (2018), our conclusion based on the present research also demonstrated that autophagy contributed to PF. Taken together, 3-MA is effective in preventing the development of PF and autophagy might be a therapeutic target for long-term PD patients.

Despite the emerging evidence showing the induction of autophagy during PF, the upstream signaling leading to autophagy activation remains unclear. It is generally known that peritoneal dialysate currently used in PD patients usually contains high glucose, advanced glycation end products (AGEs) and glucose degradation products (GDPs). In this study, we demonstrated that high glucose PDF increased TGF-β1 production. Accumulating data have indicated that TGF-β1 induces autophagy under different pathological conditions (Ding and Choi, 2014; Sureshbabu et al., 2016; Zhang et al., 2017b). TGF-β1 stimulation increases autophagosomes accumulation and autophagic flux activation, and up-regulates autophagy-related genes such as Atg5, Atg7, LC3, Beclin-1 and Death-associated protein kinase (DAPK) (Kiyono et al., 2009; Ding and Choi, 2014; Sureshbabu et al., 2016). Similarly, our *in vitro* study showed that TGF-β1 (2 ng/ml) stimulation increased autophagic activity in cultured HPMCs with the up-regulation of LC3 and Beclin-1. As a gold standard to observe autophagosome, images from TEM revealed accumulation of autophagy-related structures in TGF-β1-stimulated cells compared to starved cells. TGF-β1 has been shown to activate PI3K/Akt/mTOR pathway (Lamouille and Derynck, 2007), which is important to mediate autophagy. Therefore, high glucose PDF stimulates the production of TGF-β1 might induce autophagy by regulating PI3K/Akt/mTOR pathway. Additionally, AGEs can activate autophagy as well. Biological function of extracellular AGEs mainly depends on the interaction with receptor for AGEs (RAGE). After that, the AGEs in league together with intracellular AGEs induce oxidative stress and increase generation of reactive oxygen species (ROS) dependent or independent on protein kinase C (PKC) activity, finally leading to autophagy (Ding and Choi, 2015). Moreover, the other two pathways, ERK-DAPK-Beclin1-hVps34 and CaMKKβ-AMPK are also activated by AGEs and focus on autophagy (Xie et al., 2013). Recent study also confirmed that autophagy was highly up-regulated in peritoneal membrane from long-term PD patients, as evidenced by increased expression of LC3 and autophagosomes, which was related to PF (Wu et al., 2018). Collectively, these data suggest that long-term PD induce the activation of autophagy.

Considering that autophagy is activated in long-term PD patients during PF, it is worth to explore the possible fibrogenesis mechanisms associated with autophagy. EMT plays an initial role in the process of fibrosis and subsequent functional deterioration of the peritoneal membrane. In this regard, we determined the relationship between autophagy and EMT in this study. Our study showed that inhibition of autophagy with 3-MA decreased the expression of EMT-related makers including α-SMA, Fibronectin, and Collagen I *in vivo* and *in vitro*. Mechanistically, we further demonstrated that inhibition of autophagy with 3-MA blocks EMT by regulating TGF-β/Smad3 signaling pathway and downstream nuclear transcription factors. Consistently in the current study, activation of EMT by autophagy was also shown in hepatocellular carcinoma cells, which associated with the activation of TGF-β/Smad3 signaling (Li et al., 2013). It can be seen that in addition to the positive role of TGF-β on autophagy, up-regulated autophagy can act back on TGF-β expression as well. Hu et al. found that autophagy-mediated phosphodiesterase 4A (PDE4A) degradation could activate CAMP/PKA/CREB signaling, which contributed to TGF-β production (Hu et al., 2018). However, the relationship between autophagy and EMT is complicated, a recent study demonstrated that autophagy could promote EMT in both TGF-β-dependent and independent manner (Bao et al., 2020), which indicates that there might be other pathway involved in the 3-MA-mediated inhibition of EMT. In this respect, our current study also found that EGFR/ERK1/2 signaling was upregulated in PF rats during EMT, administrated with 3-MA significantly inhibited the activation of this signaling pathway. Zhang et al. demonstrated that the loss-of-function in ATG genes may activate EGFR signaling by driving the accumulation of activated, Krn-bound EGFR complexes in Rab11 recycling endosomes and/or at the plasm membrane, such that constitutively activating downstream ERK signaling in cancers (Zhang et al., 2019). Whether the dysfunction of autophagy may trigger hyperactivation of EGFR/ERK1/2 signaling through this mechanism during EMT in PF model is still needed further exploration. Taken together, our results suggest that autophagy might promote EMT through multiple mechanisms.

Though the mechanism is not fully clarified, the crosstalk between autophagy and inflammation has recently become an interesting topic. On the one hand, autophagy directly affects the development, homeostasis and survival of several inflammatory cells, such as macrophages, neutrophils and lymphocytes (T cells and B cells), and influencing the transcription, processing and secretion of inflammatory factors (Qian et al., 2017). On the other hand, autophagy is regulated by inflammatory cytokines as well (Wu et al., 2016b). In this study, we observed an elevation of multiple proinflammation cytokines, including MCP-1, IL-1β, and IL-6, as well as macrophage infiltration of the peritoneum in PDF rats, inhibition of autophagy with 3-MA blocked all these responses. Mechanistically, it is well-established that NF-κB signaling is a critical transduction pathway that regulates cell stress and inflammatory responses (Mendis et al., 2008). In this study, we observed that NF-κB signaling pathway was evidently activated after exposure to non-biocompatible peritoneal dialysate. Combination therapy with 3-MA down-regulated phosphorylated NF-κB. Since our previous studies have already confirmed that inflammatory response is one of the key pathologic processes involved in PF during long-term PD (Wang et al., 2016; Shi et al., 2020), thus we can draw a conclusion that autophagy contributes to PF by mediating inflammatory responses, which may associate with NF-κB pathway. In fact, although our current study suggests that autophagy can mediate the NF-κB signaling, existing studies have shown that the NF-κB signaling pathway can in turn regulate autophagy (Levine et al., 2011; Xu et al., 2021). Obviously, there is an interaction between the activation of autophagy and the NF-κB signaling pathway, the relationships in detail need to further elucidate.

Inhibition of peritoneal angiogenesis may also be a mechanism by which 3-MA prevents PF. In this study, peritoneal angiogenesis was more serious in 4.25% PDF-model group than control group, treatment with 3-MA significantly blocked the peritoneal angiogenesis as evidenced by the decreased of CD31- and CD34-positive vessels, as well as VEGF-positive endothelial cells. In consistent with our observations, Liang et al. showed that rapamycin-mediated autophagy enhanced the pro-angiogenic effect, and 3-MA effectively attenuated angiogenesis evidenced by decreased proliferation and migration of human umbilical vein endothelial cells (HUVECs), and formation of tube-like structures (Liang et al., 2018). The role of WNT/β-catenin signaling in peritoneal membrane angiogenesis has been recently clarified. Our study found that β-catenin pathway was dramatically activated in PF rats, treatment with 3-MA remarkably decreased the expression of β-catenin in fibrotic peritoneum. The activation of autophagy could augment the WNT/β-catenin signaling pathway (Liu et al., 2021), as a result, β-catenin binds to the T cell factor/lymphoid enhancer-binding factor (TCL/LEF) family in the nucleus, and changes the expression of crucial mediators of angiogenesis, such as VEGF (Liu et al., 2021). In addition, autophagy also regulates pigment epithelium derived factor (PEDF) expression, an endogenous VEGF inhibitor, and increases VEGF/PEDF ratio, promoting the formation of neovascularization (Li et al., 2019b). Collectively, 3-MA prevents peritoneal angiogenesis in PF rat model through inhibiting autophagy-mediated VEGF production, and suppressing the activation of β-catenin signaling. Autophagy may become a therapeutic target in the clinical treatment of angiogenesis for long-term PD patients.

Though the role of autophagy in PF is still controversial, our current study also has some strengths. Firstly, we determined the role of autophagy in PF using two rat models induced by 4.25% PDF and 0.1% CG, both were well-documented animal models used for studying chronic peritoneal changes (Wang et al., 2016; Xu et al., 2017), and considered to be ideal models to examine the efficacy of potential therapeutic regents for treating PF (Yoshio et al., 2004). Similar results were observed in both models, that is, autophagy promotes the development of PF, it makes the conclusion draw from the current study more convincing. Secondly, since 3-MA has not yet been approved clinically for the treatment of PF, we evaluated the effect of 3-MA on fibrosis pathological changes *in vivo* and *in vitro*, which provides the preclinical evidence for the anti-fibrotic effect of 3-MA in PF. Finally, we used two doses in exploring the anti-fibrotic effect of 3-MA and found that 30 mg/kg is more effective than 15 mg/kg. Although it is acknowledged that 3-MA is not a specific autophagy inhibitor and it is likely that a higher dosage of 3-MA may have adverse effects, our research shown that higher dosage of 3-MA (30 mg/kg) have no bad effect on normal rats. Nevertheless, efficacy and safety of 3-MA for the treatment of PF are still need to further study.

In conclusion, we confirmed that autophagy promotes PF, and pharmacological blockade of autophagy protects against peritoneal injury. Autophagy was highly activated in fibrotic peritoneum from two PF rat models induced by 4.25% PDF and 0.1% CG, and in cultured HPMCs after TGF-β1 stimulation. Treatment with 3-MA significantly prevents PF through inhibiting EMT, inactivating fibrogenesis signaling pathways, down-regulating inflammation, and reducing peritoneal angiogenesis. Therefore, autophagy inhibition may have a potential therapeutic benefit for long-term PD patients.

## Data Availability

The original contributions presented in the study are included in the article/Supplementary Material, further inquiries can be directed to the corresponding author.

## Supplementary Material

Supplementary Figure S1 Administration of 3-MA inhibits autophagy and attenuates peritoneal fibrosis in 0.1% CG -induced rat model. **(A, B)** Masson’s trichrome staining of the peritoneum and its positive area. **(C, D)** Sirius Red staining of the peritoneum and its positive area. **(E)** Peritoneum tissue lysates were subjected to immunoblot analysis with specific antibodies against LC3, Beclin-1 and GAPDH. **(F)** Expression levels of LC3II and Beclin-1 were quantified by densitometry and normalized with LC3I and GAPDH respectively. Data are represented as the mean ± SEM. Means with different superscript letters are significantly different from one another (*p* < 0.05). All scale bars = 100 μm.

Supplementary Figure S2 Inhibition of autophagy with 3-MA blocks EMT in 0.1% CG -induced rat model. **(A)** Peritoneum tissue lysates were subjected to immunoblot analysis with specific antibodies against α-SMA, Fibronectin, Collagen I, E-cadherin and GAPDH. (**B–E**) Expression levels of α-SMA, Fibronectin, Collagen I and E-cadherin were quantified by densitometry and normalized with GAPDH. Data are represented as the mean ± SEM. Means with different superscript letters are significantly different from one another (*p* < 0.05).

Supplementary Figure S3 Delayed administration of 3-MA inhibits TGF-β1- induced EMT in HPMCs. **(A)** Diagram depicts treatment scheme with 3-MA. **(B)** Cell lysates were subjected to immunoblot analysis with specific antibodies against α-SMA, Collagen I, E-cadherin and GAPDH. **(C–E)** Expression levels of α-SMA, Collagen I and E-cadherin were quantified by densitometry and normalized with GAPDH. Data are represented as the mean ± SEM. Means with different superscript letters are significantly different from one another (*p* < 0.05).

Supplementary Figure S4 siRNA-mediated silencing of Beclin-1 inhibits TGF-β1 induced activation of Smad3/ERK1/2/STAT3 pathway in HPMCs (**A**) Cell lysates were subjected to immunoblot analysis with specific antibodies against Beclin-1, α-SMA, Collagen I and GAPDH. **(B)** Expression levels of Beclin-1, α-SMA and Collagen I were quantified by densitometry and normalized with GAPDH. **(C)** Cell lysates were subjected to immunoblot analysis with specific antibodies against p-Smad3, Smad3 and GAPDH. **(D)** Expression level of p-Smad3 was quantified by densitometry and normalized with Smad3. **(E)** Cell lysates were subjected to immunoblot analysis with specific antibodies against p-ERK1/2, ERK1/2 and GAPDH. **(F)** Expression levels of p-ERK1/2 was quantified by densitometry and normalized with ERK1/2. **(G)** Cell lysates were subjected to immunoblot analysis with specific antibodies against p-STAT3, STAT3 and GAPDH. **(H)** Expression levels of p-STAT3 was quantified by densitometry and normalized with STAT3. Data are represented as the mean ± SEM. Means with different superscript letters are significantly different from one another (*p* < 0.05).

## Glossary

AGEs: advanced glycation end products
CG: chlorhexidine gluconate
CTGF: connective tissue growth factor
DAPK: Death-associated protein kinase
DMSO: dimethyl sulfoxide
ECM: extracellular matrix
EGFR: epidermal growth factor receptor
ELISA: enzyme-linked immunosorbent assay
ERK1/2: extracellular signal–regulated kinase 1/2
EMT: epithelial to mesenchymal transition
ESRD: end-stage renal disease
FBS: fetal bovine serum
FFPE: Formalin-Fixed Paraffin-Embedded
GAPDH: glyceraldehyde 3-phosphate dehydrogenase
GDPs: glucose degradation products
HG: high glucose
HUVECs: human umbilical vein endothelial cells
HPMCs: human peritoneal mesothelial cells
IL-1β: interleukin-1β
IL-6: interleukin-6
LC3: light chain 3
LEF: lymphoid enhancer-binding factor
MMT: mesothelial to mesenchymal transition
MCP-1: monocyte chemoattractant protein-1
3-MA: 3-methyladenine
NF-κB: nuclear factor-κB
PKC: protein kinase C
PDE4A: phosphodiesterase 4A
PEDF: pigment epithelium derived factor
PF: peritoneal fibrosis
PD: peritoneal dialysis
PDF: peritoneal dialysate fluid
ROS: reactive oxygen species
STAT3: signal transducer and activator of transcription 3
α-SMA: α-smooth muscle actin
TCL: T cell factor
TEM: transmission electron microscope
TNF-α: tumor necrosis factor-α
TGF-β: transforming growth factor-β
TGF-βRI: type I TGF-β receptor
VEGF: vascular endothelial growth factor
