# Supplementary figures and images for: Blockade of Autophagy Prevents the Development and Progression of Peritoneal Fibrosis
Source: Front Pharmacol. 2021 Aug 23;12:724141. doi: 10.3389/fphar.2021.724141 (PMC8419262; doi:10.3389/fphar.2021.724141)

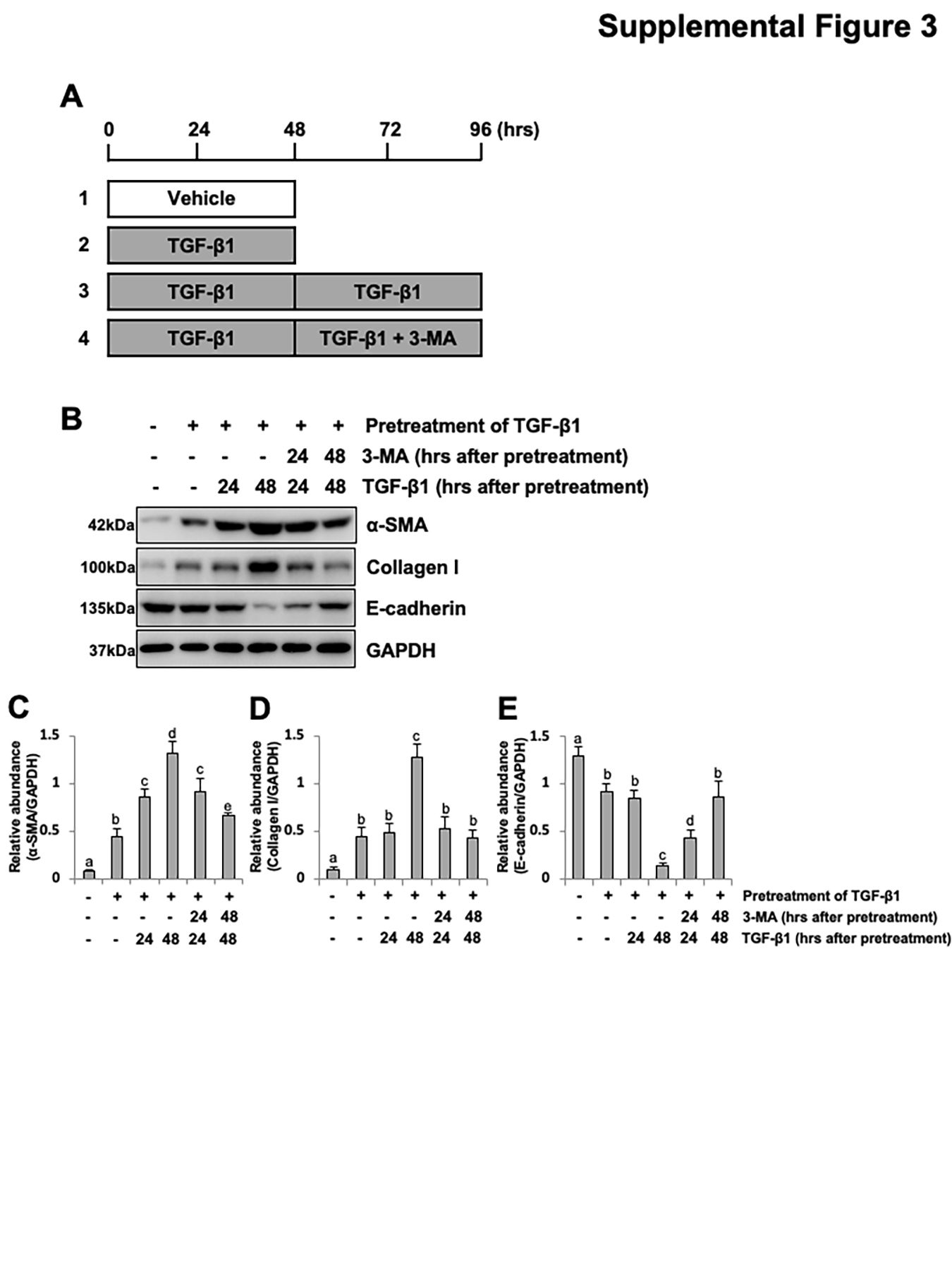

Supplement: Supplementary file 1 [file image3.tiff]

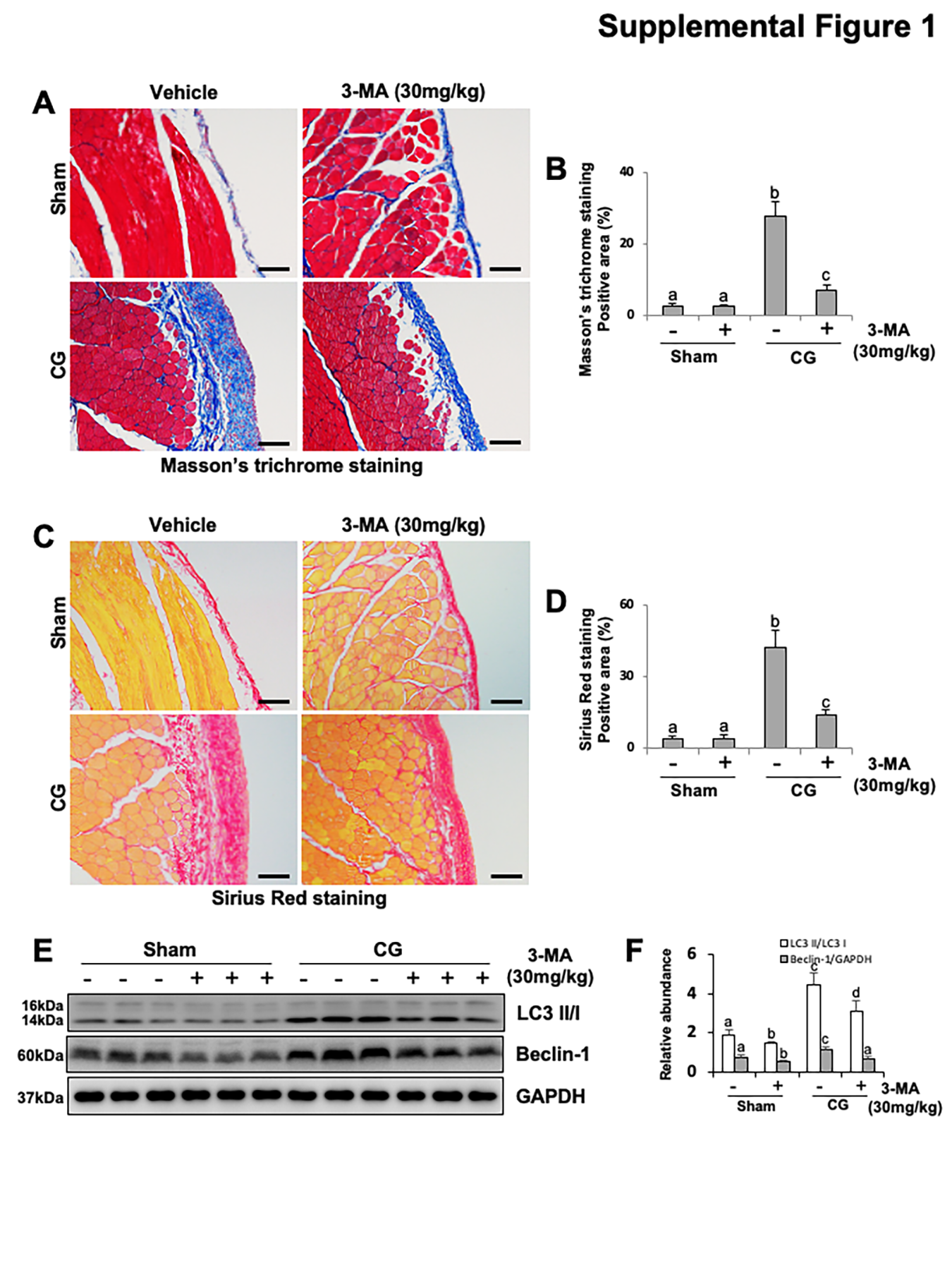

Supplement: Supplementary file 2 [file image1.tiff]

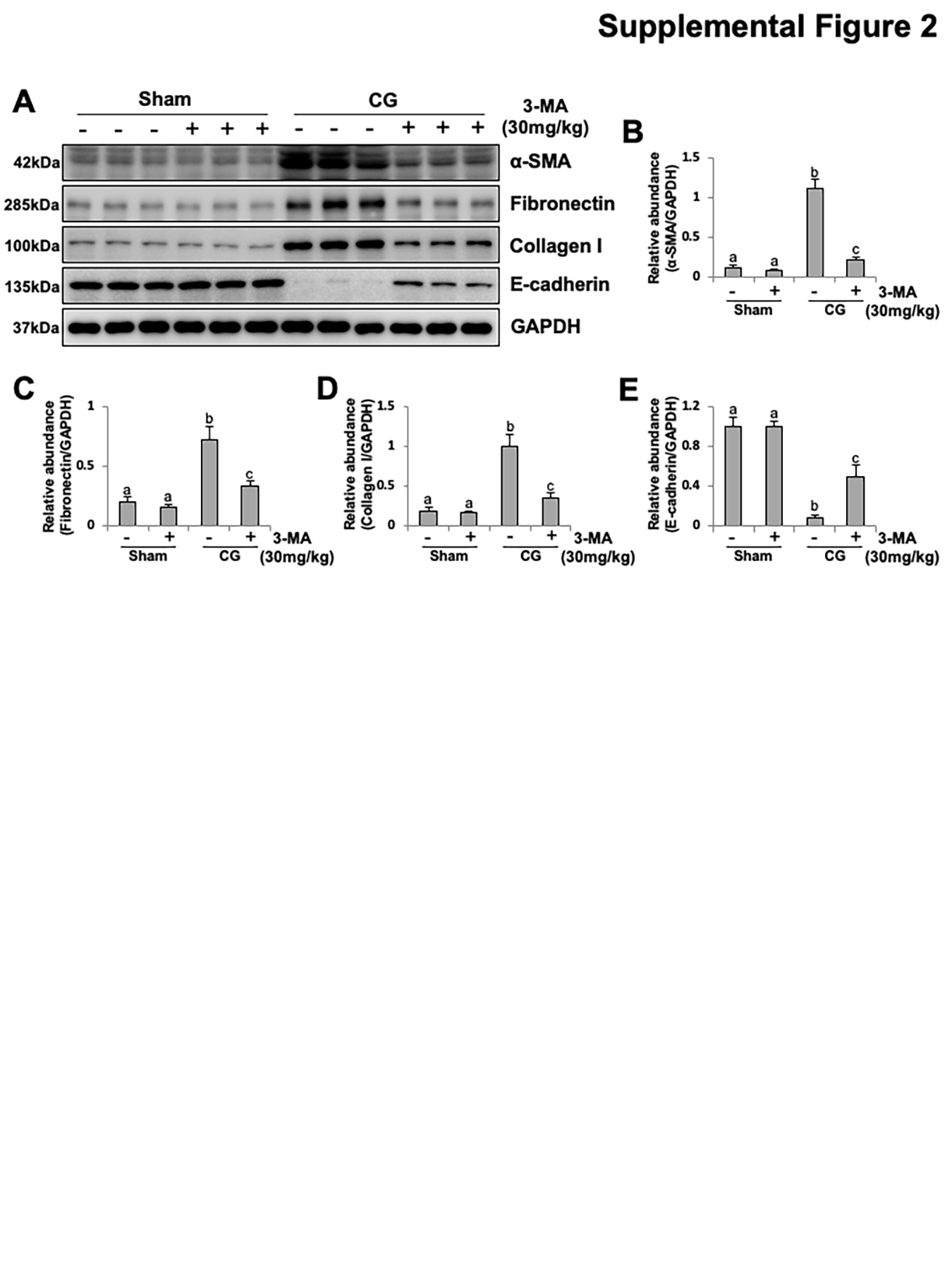

Supplement: Supplementary file 3 [file image2.tiff]

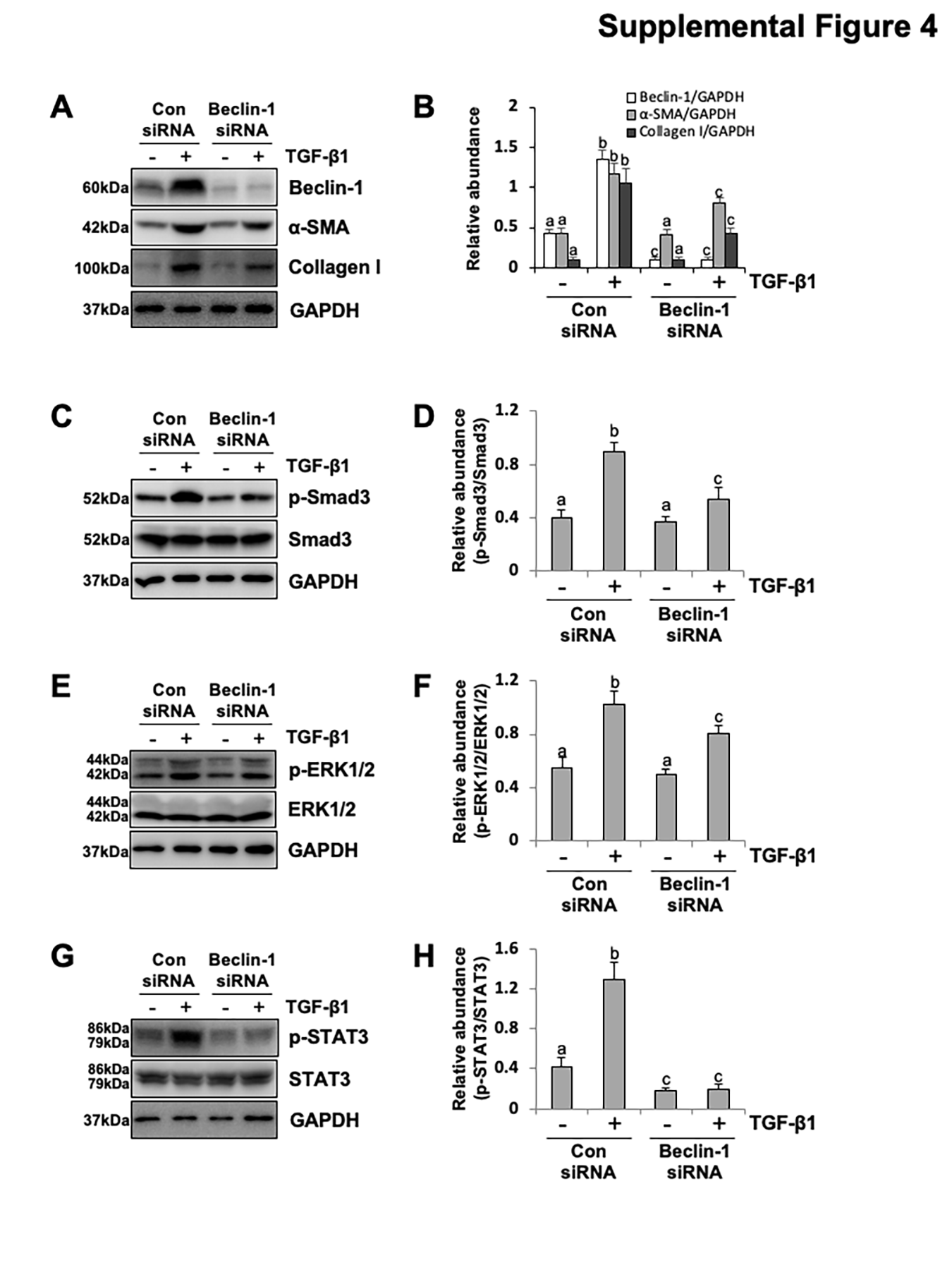

Supplement: Supplementary file 4 [file image4.tiff]
